# Building a regional pediatric asthma learning health system in support of optimal, equitable outcomes

**DOI:** 10.1002/lrh2.10403

**Published:** 2023-12-11

**Authors:** Andrew F. Beck, Michael Seid, Karen M. McDowell, Mfonobong Udoko, Susan C. Cronin, Dimitrios Makrozahopoulos, Tricia Powers, Sonja Fairbanks, Jonelle Prideaux, Lisa M. Vaughn, Elizabeth Hente, Sophia Thurmond, Ndidi I. Unaka

**Affiliations:** ^1^ Division of General & Community Pediatrics Cincinnati Children's Cincinnati Ohio USA; ^2^ Division of Hospital Medicine Cincinnati Children's Cincinnati Ohio USA; ^3^ James M. Anderson Center for Health Systems Excellence Cincinnati Children's Cincinnati Ohio USA; ^4^ Michael Fisher Child Health Equity Center Cincinnati Children's Cincinnati Ohio USA; ^5^ Office of Population Health Cincinnati Children's Cincinnati Ohio USA; ^6^ Department of Pediatrics University of Cincinnati College of Medicine Cincinnati Ohio USA; ^7^ Division of Pulmonary Medicine Cincinnati Children's Cincinnati Ohio USA; ^8^ Division of Emergency Medicine Cincinnati Children's Cincinnati Ohio USA; ^9^ Qualitative Methods & Analysis Collaborative Cincinnati Children's Cincinnati Ohio USA; ^10^ Criminal Justice, & Human Services University of Cincinnati College of Education Cincinnati Ohio USA; ^11^ UC Health Cincinnati Ohio USA; ^12^ Department of Information Services Cincinnati Children's Cincinnati Ohio USA

**Keywords:** asthma, health equity, learning health systems, pediatrics, population health

## Abstract

**Introduction:**

Asthma is characterized by preventable morbidity, cost, and inequity. We sought to build an Asthma Learning Health System (ALHS) to coordinate regional pediatric asthma improvement activities.

**Methods:**

We generated quantitative and qualitative insights pertinent to a better, more equitable care delivery system. We used electronic health record data to calculate asthma hospitalization rates for youth in our region. We completed an “environmental scan” to catalog the breadth of asthma‐related efforts occurring in our children's hospital and across the region. We supplemented the scan with group‐level assessments and focus groups with parents, clinicians, and community partners. We used insights from this descriptive epidemiology to inform the definition of shared aims, drivers, measures, and prototype interventions.

**Results:**

Greater Cincinnati's youth are hospitalized for asthma at a rate three times greater than the U.S. average. Black youth are hospitalized at a rate five times greater than non‐Black youth. Certain neighborhoods bear the disproportionate burden of asthma morbidity. Across Cincinnati, there are many asthma‐relevant activities that seek to confront this morbidity; however, efforts are largely disconnected. Qualitative insights highlighted the importance of cross‐sector coordination, evidence‐based acute and preventive care, healthy homes and neighborhoods, and accountability. These insights also led to a shared, regional aim: to equitably reduce asthma‐related hospitalizations. Early interventions have included population‐level pattern recognition, multidisciplinary asthma action huddles, and enhanced social needs screening and response.

**Conclusion:**

Learning health system methods are uniquely suited to asthma's complexity. Our nascent ALHS provides a scaffold atop which we can pursue better, more equitable regional asthma outcomes.

**Abbreviations**
ALHS: Asthma Learning Health System
CDC: Centers for Disease Control and Prevention
ED: Emergency Department
EHR: Electronic Health Record
GLA: Group‐Level Assessment
QI: Quality Improvement

## 1 INTRODUCTION

Asthma in children is associated with avoidable morbidity and unacceptable inequity.^1^ Asthma‐related hospitalizations result in children and families experiencing trauma from acute illness,^2^, ^3^ and, often, missed school and work. Asthma is among the most expensive pediatric conditions, requiring care in pediatric intensive care units, inpatient wards, and emergency departments (ED). While clinical outcomes have improved for patients who reach hospitals,^4^ preventing asthma exacerbations and addressing inequities^1^, ^5^, ^6^ requires robust population health approaches not yet realized in many regions, including our own. Despite best intentions and some success,^1^, ^4^, ^7^ asthma‐relevant community services, clinical care, and research often remain siloed and fragmented. At Cincinnati Children's, there are gaps between primary and subspecialty care and between inpatient, ED, and ambulatory settings. There are further gaps between the healthcare system and schools, pharmacies, public health departments, community service providers, community leaders, and parents.

A “network organization” does not require a central decision‐making body to allocate effort and resources. In complex, dynamic environments, no single person or entity has all the information necessary to make such decisions. Instead, a network organization makes it possible for individuals or teams to identify needs, form teams, share power,^8^ and collaborate at scale in pursuit of shared, common aims.^9^, ^10^, ^11^ This organizational architecture is used in many industries and sectors, from social media to ride sharing, supply chain management^12^ to the military.^10^, ^13^ We have applied such architecture to learning health systems,^14^, ^15^, ^16^ demonstrating their effectiveness in spurring collective action, accelerating research, and improving outcomes.^17^, ^18^, ^19^, ^20^, ^21^, ^22^, ^23^ This application, to our knowledge, has not yet been extended to asthma.

We, therefore, sought to build a pediatric Asthma Learning Health System (ALHS) to coordinate regional asthma activities. We hypothesized that this network organizational architecture would enable multiple clinical and community service providers, and families, to align and act. Here, we describe the first phase of our ALHS experience, mapping of the current system of asthma care, and building of infrastructure to run and optimize ALHS.

## 2 METHODS

### 2.1 Context

ALHS was built to serve children living in Hamilton County, the population center for metropolitan Cincinnati. Approximately 190 000 youth reside in Hamilton County; we estimate ~30 000 have asthma. We convened clinical and operational leaders from Cincinnati Children's, a parent partner, and community leaders in August 2021 to rethink our asthma strategy. The session focused on building empathy and understanding for the asthma experience and discussing how a collaborative, learning health system approach could be useful. The session resulted in a successful proposal to the Cincinnati Children's Research Foundation to fund ALHS, which was formally launched in March 2022.

Soon thereafter, we started with an “environmental scan”^24^ to catalog regional asthma‐related efforts. We connected with asthma‐relevant clinical and research divisions at Cincinnati Children's (eg, pulmonary, allergy, hospital medicine, general pediatrics) to identify approaches to clinical care (eg, order sets, note templates, care guidelines), research studies, quality improvement (QI) initiatives, key performance indicators, and community partnerships. In parallel, we identified community‐serving organizations across relevant sectors (eg, housing, policy/government, education, social service). We characterized organizations using publicly‐available information (eg, website, annual report), defining services offered, funding and fee structure, geographic reach, and existing connections to Cincinnati Children's.

### 2.2 Descriptive epidemiology

We used data from the Cincinnati Children's electronic health record (EHR) to quantitatively describe variations in care and outcomes for youth with asthma across the county. We focused primarily on acute service utilization, as we have near complete market penetration for hospital‐based care. Asthma‐related encounters were identified using ICD‐10 codes: J45 in the first diagnosis position; or J00, J06, J30, J31, J96.0, J96.9, R05, R06, or R09.02 in the first position with J45 in a latter position. We focused primarily on hospitalizations but also assessed ED and urgent care visits. To calculate rates, numerators were the number of encounters within a given period; denominators were the number of children and adolescents <18 years of age. We used this denominator given its availability from the U.S. Census and to align with national estimates available from the Centers for Disease Control and Prevention (CDC).^25^

We disaggregated rates across demographic groups and geographic areas. For example, we calculated rates for Black and non‐Black youth, using self‐ or parent‐reported race within the EHR. We did so to unearth inequities, cognizant that race exists as a sociopolitical construct. We also calculated hospitalization rates for neighborhoods, using geocoded home addresses and locally‐defined neighborhood boundary shapefiles. We evaluated asthma encounters retrospectively while developing capabilities to track encounters prospectively. We mapped asthma encounters atop background information that varied by either time (eg, viral infections, air quality) or space (eg, area‐based measures, or geomarkers, like traffic‐related pollutants, and poverty).^26^, ^27^, ^28^, ^29^ We did so to identify patterns and bring more partners into ALHS.

Quantitative data were complemented by qualitative data, obtained to understand facilitators and barriers key members of the system face in achieving their goals^30^ and to execute a priority‐setting process.^31^ A purposeful sample of families/caregivers of youth with asthma, clinicians, healthcare professionals, researchers, and representatives of community organizations was recruited via directed outreach, distributed flyers, and presentations to frontline clinical and community service providers. We held three ~2‐hour virtual group sessions with participants. The first two sessions used a group‐level assessment (GLA) format, a participatory large‐group method in which participants respond to prompts and collaborate to analyze patterns in responses.^32^ The third session was a focus group. Prompts and questions were constructed by members of the ALHS core team, with input from partners inside and outside the hospital. Topics emphasized community assets and gaps; family experience with care; and situational awareness to inform local decisions/needs (Table S1.). Participants engaged in real‐time thematic analysis during each session, facilitated by qualitative methods experts.

### 2.3 Designing ALHS aims, drivers, measures, and prototype interventions

We completed two “design sessions” to share results of the environmental scan, and quantitative and qualitative insights, and co‐construct aims, drivers, measures, and prototype interventions. Our first session was conducted virtually in August 2022. A Cincinnati City Councilman provided a call for collective action. This “ignite talk” was followed by a brief depiction of regional pediatric asthma. All‐group and break‐out sessions enabled attendees to become acquainted with one another and to hear, and discuss, preliminary results from the environmental scan and quantitative/qualitative analyses. To accelerate discussion, we shared asthma “personas” developed from descriptive epidemiology (eg, children with comorbidities, school‐aged children, those from neighborhood “hot‐spots”). Before the end of the day, attendees moved toward identification of a shared global aim via a “newspaper headline” exercise. Attendees were asked “If we were successful, what would the headline read in the Cincinnati Enquirer in 1, 5, or 10 years?” Attendees were also asked to consider what it would take to catalyze alignment and success. This first design session resulted in a shared global aim (“newspaper headline”) and a working ALHS structure.

Our second design session was conducted in‐person in April 2023. Many attendees participated in the first session; others were newly welcomed into ALHS. Two high school students provided the “ignite talk,” discussing the ways asthma has influenced their lives. An empathy exercise followed, pushing attendees to consider asthma from a perspective different from their own (ie, physician from perspective of parent; high school principal from the perspective of a high school student). The second design session resulted in a key driver diagram, complete with global and SMART aims, population (including scalable unit and scale plan), drivers, and priority interventions.

### 2.4 Ethical considerations

Pertinent portions of ALHS design work were reviewed and approved by the Cincinnati Children's Institutional Review Board.

## 3 RESULTS

### 3.1 Initial planning session

Seventeen individuals participated in an August 2021 planning session focused on how network organizational architecture could equitably improve the health of regional children with asthma over the next 5–10 years. This session identified a need for an environmental scan and bolstered descriptive epidemiology. Attendees aligned on the pursuit of a more detailed depiction of current state, plan for future state, and design strategy for enhanced coordination and action.

### 3.2 Environmental scan

**FIGURE 1:**
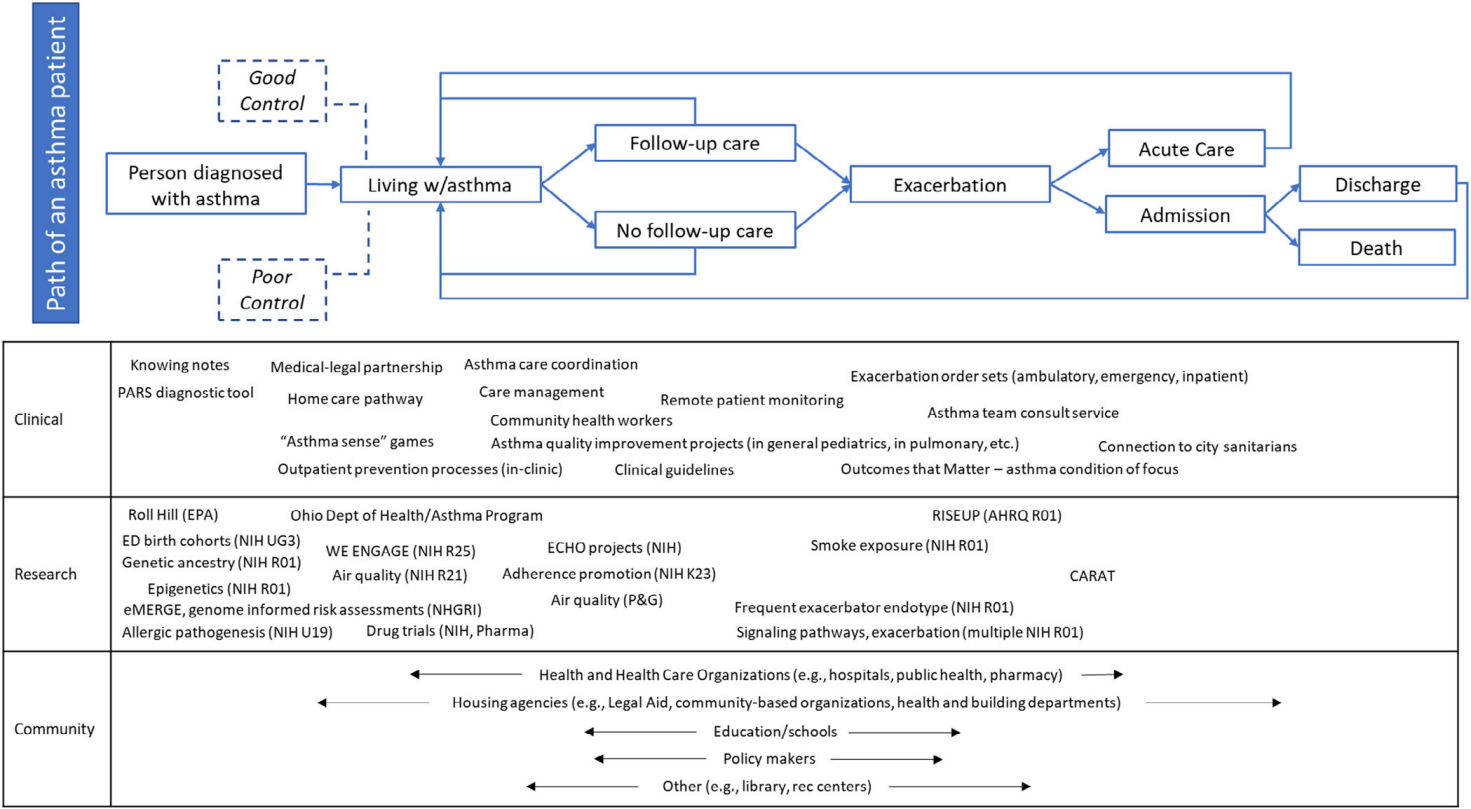
Environmental scan results, including clinical, research, and community‐based projects and teams with an asthma focus.

The environmental scan produced an inventory of activities across clinical, research, and community domains. Within the clinical domain, we identified asthma order sets, symptom assessment scores and algorithms, and note templates. For research, we identified principal investigators, collaborators, aims (research or improvement), population, funding source, data collected, and preliminary results. We also captured various definitions of asthma patients and encounters, measures tracked routinely, and links to existing dashboards or reports used to calculate key performance indicators. For community, we identified asthma‐relevant resources and supports. We found a breadth of clinical, research, and community activities, though largely disconnected from one another. Figure 1 maps identified clinical, research, and community‐based activities to the “path of an asthma patient.” This helped us identify areas of strength, areas of opportunity, and priorities for building alignment.

### 3.3 Descriptive epidemiology

**FIGURE 2:**
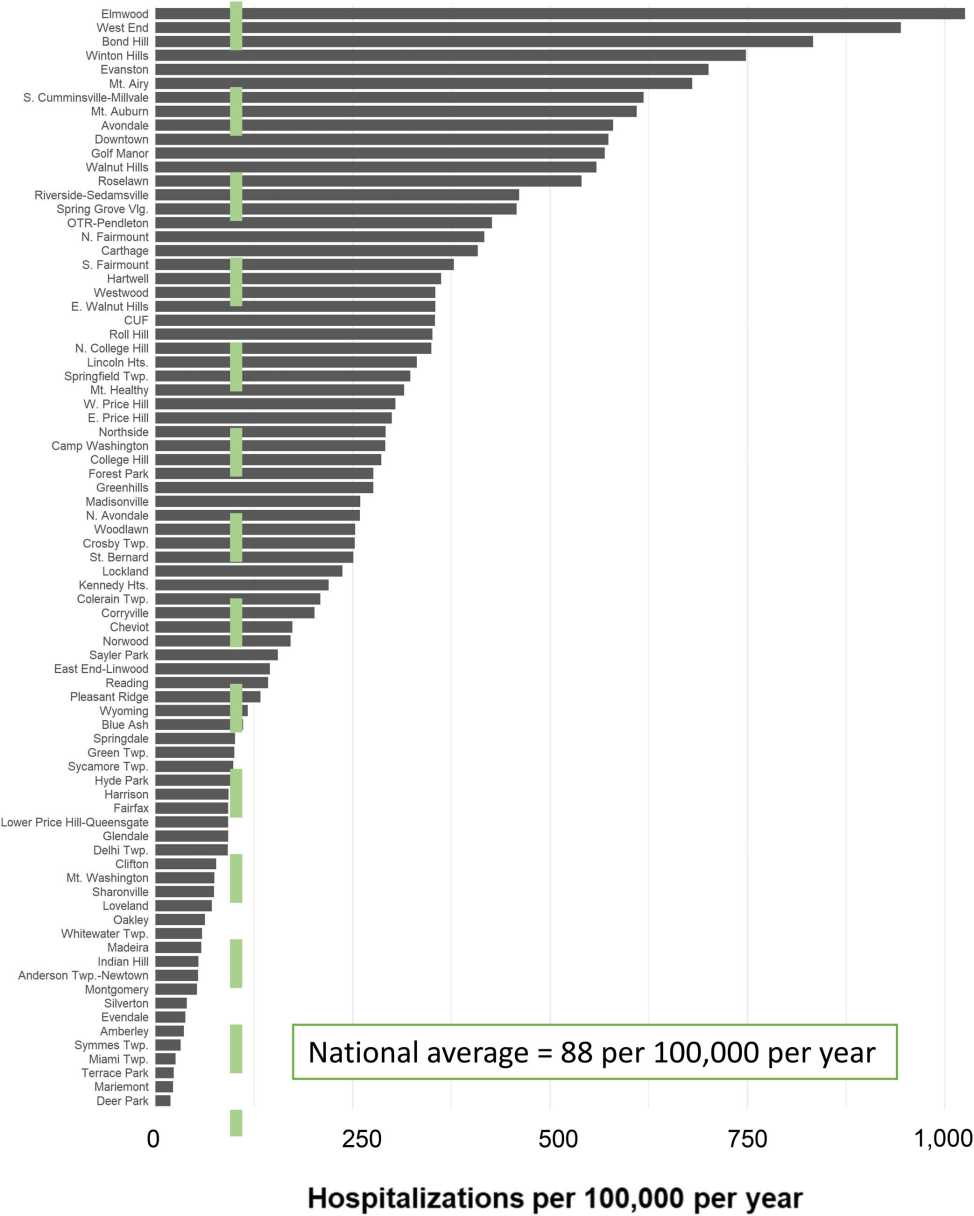
Depiction of variability in asthma hospitalization outcome by Hamilton County, Ohio neighborhood, measured as a rate per 100 000 children <18 years per year, using data from 2017 to 2021. The national asthma hospitalization for children was obtained from estimates published by the Centers for Disease Control and Prevention.^25^

Per the CDC, the national asthma hospitalization rate for children and adolescents <18 years is 88 hospitalizations per 100 000 children per year.^25^ Using data from our EHR, we calculated the Hamilton County hospitalization rate to be 263 per 100 000, three‐times the national average. The in‐county hospitalization rate for Black youth was 599 per 100 000; the rate for non‐Black youth was 117. Asthma accounts for nearly 20% of the Black/non‐Black gap across all causes of hospitalization for children in the county, nearly 30% of the gap in hospitalizations related to physical illnesses. Closing this equity gap would require us to prevent ~300 annual asthma admissions for Black patients. Moreover, there is significant variability across Hamilton County neighborhoods, some with asthma hospitalization rates many times the national average (Figure 2). Data such as these were motivating for those entering our growing ALHS.

Twenty‐two participants attended the three qualitative sessions. Participants identified themselves as mothers (n = 10), a grandmother (n = 1), physicians (attendings, fellows, and residents; n = 6), other healthcare and community professionals (asthma educator, clinical manager, care manager, and community health worker; n = 4), and a health systems leader (n = 1). Nineteen identified as female, three as male. Participants identified as Black (n = 11), White (n = 10), and other (n = 1).

During the first GLA session, 11 participants identified 21 main ideas grouped into three salient themes. First, participants voiced a need for improved access to medications inside and outside the home (ie, schools, daycare), reduced barriers to obtaining refills from the pharmacy, and a better understanding of the “why” of medication regimens. Second, participants expressed a desire for asthma supports (and education) to extend to educators, childcare providers, and other family members (eg, grandparents, siblings). Third, participants, particularly parents, highlighted the constant fear and stress that accompanies having a child with asthma, given the unpredictable nature of triggers and difficulties controlling symptoms. These emotions were often coupled with a desire to live a “normal life” and not one characterized by fear of asthma controlling their lives.

Five participants attended the second GLA session. Participants underscored the idea that asthma care should not use a one‐size‐fits‐all approach. They recommended ongoing support via easily accessible, high‐quality resources tailored to child and family needs. Participants also stressed a need for continuous education that could be adapted to a child's unique developmental timeline and applied across a child's care environment (eg, home, clinic, school). Coordination of medical and non‐medical individuals in a child's life was deemed essential. Finally, participants suggested accountability for optimal regional outcomes must be shared. That said, a point person (or persons) must ensure that shared accountability does not create more cracks into which patients (and populations) might fall.

Six participants attended the third session, a focus group. Participants identified the importance of an adapted chronic care model,^33^ one that helps those caring for a child with asthma recognize the importance of consistency—a “long haul” mentality. Participants detailed how hard it can be to manage asthma amidst the challenges of daily life, particularly when living in poverty. As such, participants indicated a need for a customized care approach, providing the “right” support for each family at the “right” time and place. Participants identified the need for prevention resources (eg, apps) that could be shared across settings (eg, schools, grandparents' house) in easy‐to‐use, accessible ways.

Information from all three sessions was then compiled. Five overarching themes emerged:

1. Locating and accessing appropriate, tailored resources is a “pain point” for families, especially since many are unaware of tools that could prevent asthma exacerbations and improve quality‐of‐life.
2. Many families are unaware of free or low‐cost asthma resources (eg, dust mite covers).
3. Resources and information must be user‐friendly and easily accessible by all involved in a child's life, including those outside the home (eg, schools).
4. Many families desire comprehensive care plans prioritizing day‐to‐day management.
5. Healthcare providers view asthma education as a priority but worry about overburdening families with too much information at once.

### 3.4 Shared ALHS aims, drivers, measures, and prototype interventions

**FIGURE 3:**
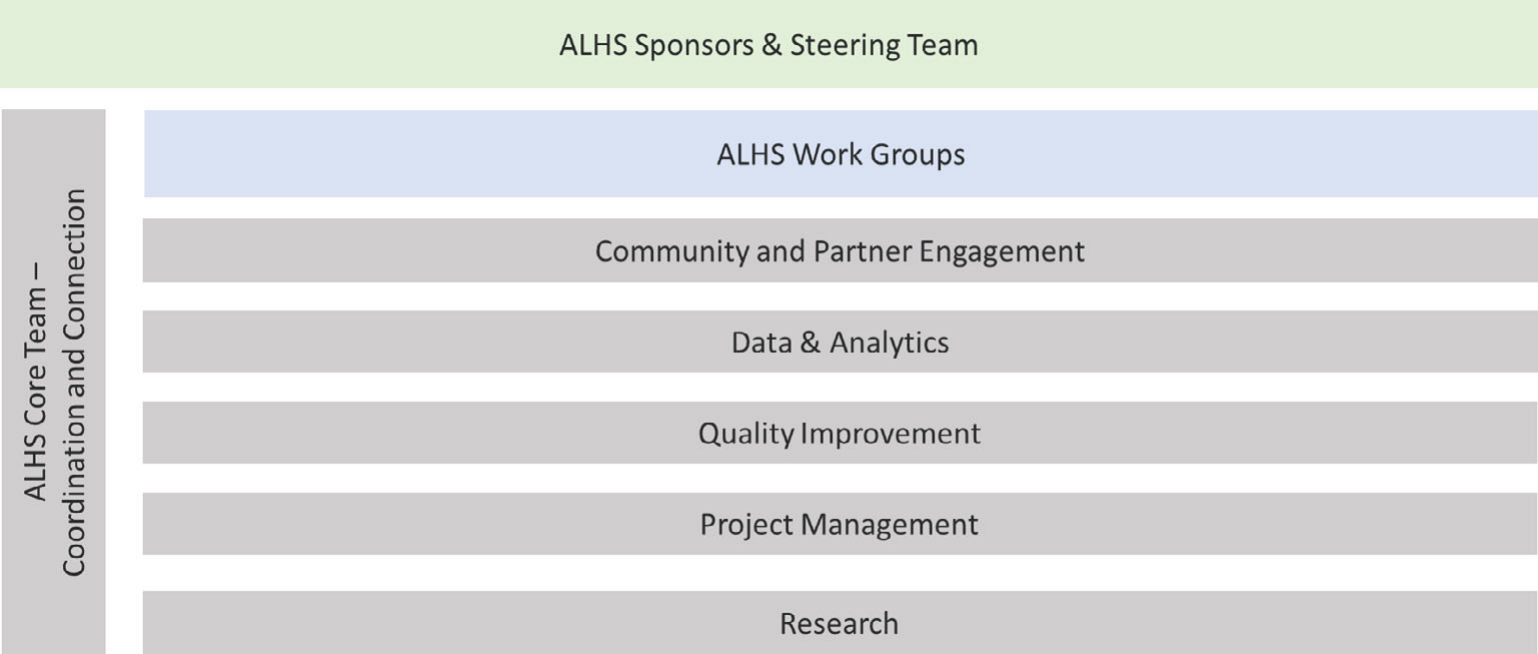
Asthma Learning Health System (ALHS) organizational structure.

Environmental scan and descriptive epidemiology data were used across two design sessions which sought to define aims, drivers, measures, and prototype interventions. A total of 65 individuals representing healthcare, school, city government, city and county health departments, housing, and environmental health sectors participated in the first, 6‐hour virtual session in August 2022. Two caregivers of youth with asthma also attended. Attendees agreed on the ALHS global aim, emergent from the “newspaper headline” exercise: “Cincinnati: the best and healthiest place for kids with asthma to live, grow, play, and THRIVE!” The session also informed the establishment of an ALHS leadership structure. We created a Sponsors Committee and Steering Team, comprised of leaders in healthcare and community agencies, to provide direction for ALHS operations, mitigation of barriers, and identification of areas for collaboration (Figure 3). Work teams began huddling to consider when and where to alter existing care processes and when to test innovations. Teams were supported by community engagement specialists, data analysts, QI specialists, project managers, and research associates. A core team coordinated and connected efforts.

**FIGURE 4:**
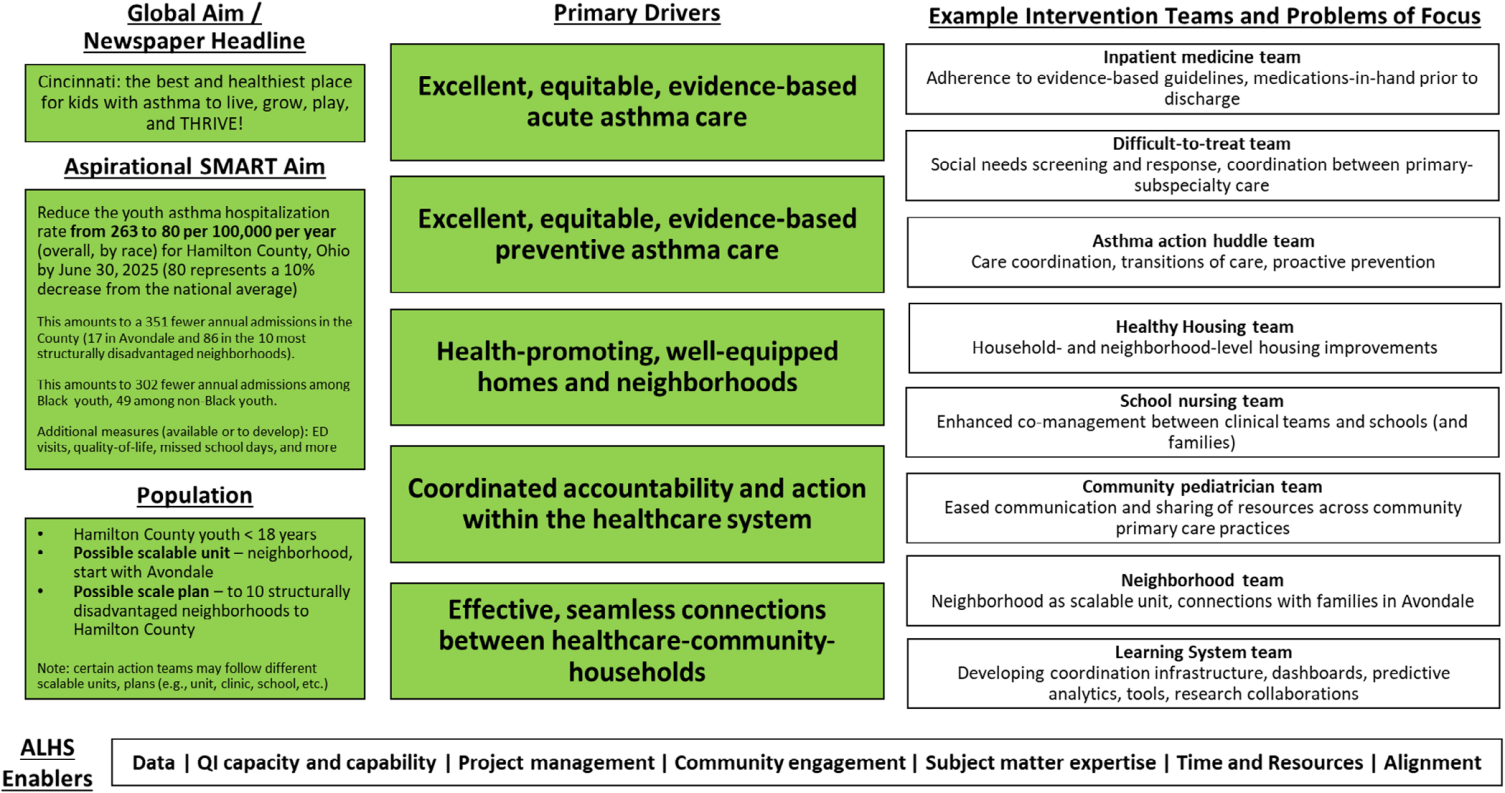
Asthma Learning Health System Key Driver Diagram. The global aim is the output of our first design session's “newspaper headline” exercise. The aspirational SMART aim is focused on hospitalizations. However, we recognize that this aim is both aspirational and related to other measures that are not yet as feasible to measure at a population scale. Such additional, “under the surface,” measures are listed below the hospitalization‐focused SMART aim. ED, Emergency Department; SMART, Specific, Measurable, Achievable, Relevant, Time‐Bound.

Sixty‐eight individuals, including parents and patients, healthcare providers, and officials from school, city government, city and county health departments, housing, and environmental health sectors, participated in the second, 4‐hour in‐person design session in April 2023 which sought to build the bridge toward action. The key driver diagram in Figure 4 displays our global aim alongside a SMART aim and population refined during this session. The group agreed on an aim to decrease the asthma‐related hospitalization rate for Hamilton County, Ohio to 10% less than the national average, from 263 to 80 per 100 000 per year. Equity was central to this aim, explicitly focusing on the overall population and a population disaggregated by race and neighborhood. Absolute numbers were added—equating to 351 fewer annual in‐county admissions (302 among Black youth, and 49 among non‐Black youth) if our aim was achieved. The group also saw “neighborhood” as a possible scalable unit, a representative slice of the population.^34^ As such, absolute numbers were added for structurally‐disadvantaged neighborhoods experiencing high rates of asthma hospitalizations. We agreed upon clinic‐ and community‐level drivers‐excellent, equitable, evidence‐based acute and preventive care and health‐promoting homes and neighborhoods—as well as organizational drivers—coordinated accountability and effective, seamless connections. This reflects the fact that a key ALHS function is to ensure necessary resources are coordinated and deployed to where they are most needed. Finally, the driver diagram includes a growing number of work teams focused on solutions and innovations.

Although we are still early in the translation of infrastructure and theory to action, we highlight three activities currently being implemented: (1) a shared asthma dashboard and approach to population‐level pattern recognition; (2) weekly multidisciplinary asthma action huddles; and (3) enhanced social needs screening and response. As possible, each activity has been developed and is continuously optimized with diverse input from key members of the asthma system.^35^

**FIGURE 5:**
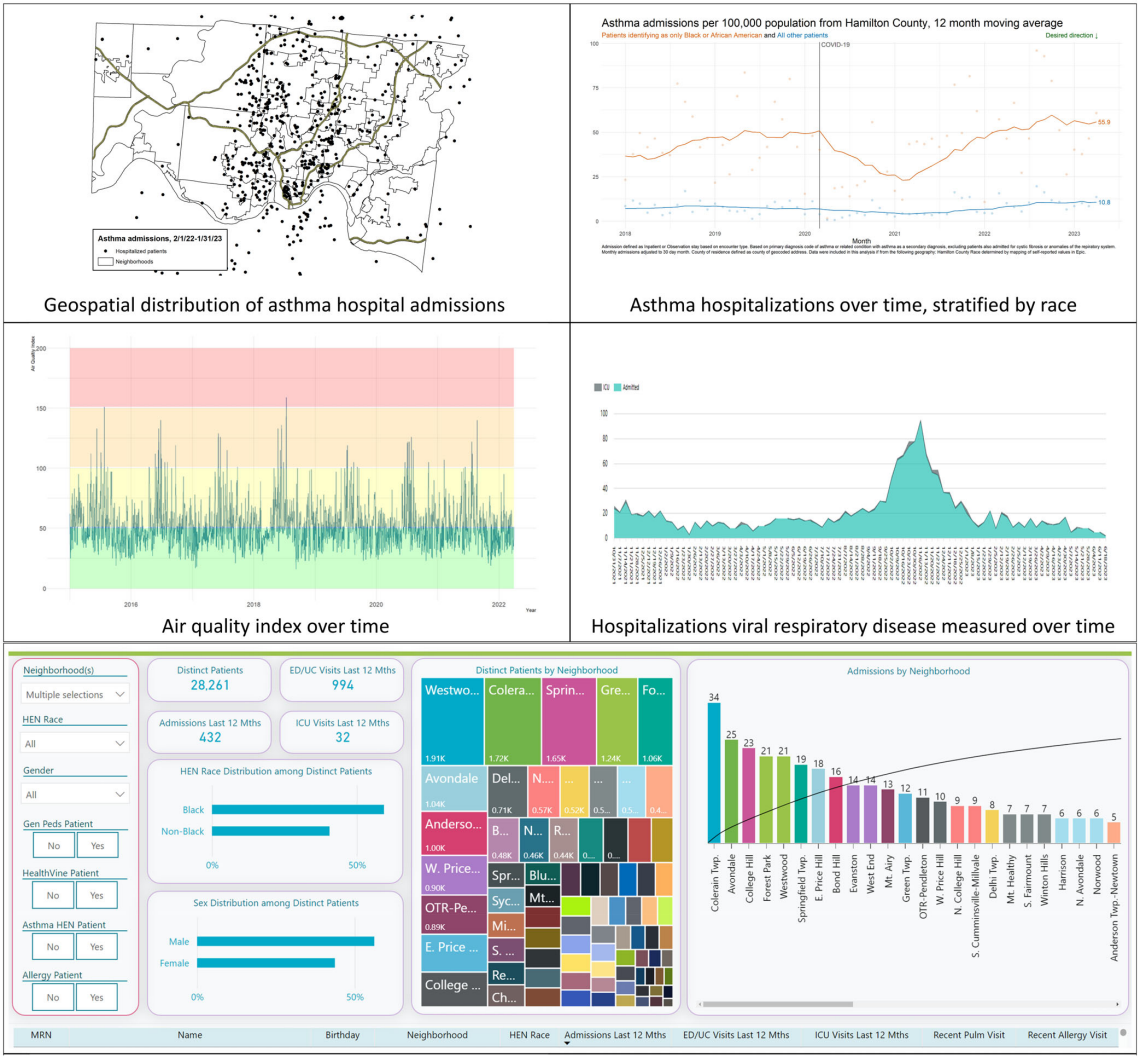
Prototype data dashboard and data tool used to provide shared situational awareness for members of the asthma system, depicting geospatial distribution of asthma hospital admissions, racial inequities, and potential triggers like air quality and viral infections, alongside the interactive bottom pane which enables sorting by key sociodemographic and geographic factors. The bottom pane enables real‐time sorting based on clinic (gen peds = Cincinnati Children's Primary Care; Asthma HEN = Pulmonary Medicine; Allergy = Allergy Clinic), payer (HealthVine is a Medicaid Accountable Care Organization). ED, Emergency Department; HEN‐Health Equity Network; UC, Urgent Care.

We created dashboard prototypes to ease population‐level pattern recognition for asthma‐related risks and outcomes. This includes visualizations illustrating geospatial distributions of hospitalizations, hospitalizations measured over time, hospitalizations stratified to depict inequities, geomarkers related to potentially‐relevant exposures (eg, socioeconomic deprivation, nearby roadway density, greenspace, air quality, pollen, mold, ozone, nitrogen dioxide), and time‐varying hospitalizations related to viral infections (Figure 5).^7^, ^28^, ^36^ This approach has already led to the identification of problem apartment complexes in neighborhoods with some of the highest asthma hospitalization rates.^37^ Figure 5 also displays a screenshot of an interactive asthma tool developed using data from the EHR. We can manipulate data using this tool to identify pockets of patients cared for in different clinical settings (eg, Cincinnati Children's primary care or subspecialty clinics), with different payers, of different ages, or with a history of frequent exacerbation. We see a great opportunity to continue to add to this dashboard as additional data become available—eg, measures like missed school, medication adherence, pulmonary function tests, biomarkers, etc. The tool also includes run‐charts related to asthma‐related hospitalizations and ED visits. Different charts depict utilization rates at various geographies (ie, Hamilton County, individual neighborhoods). We stratify charts, when appropriate, by race and payer to enable an evaluation of inequities.

This dashboard and data tool are pertinent to our second innovation, a weekly asthma action huddle. The huddle, attended by a multidisciplinary team of physicians, nurses, social workers, care managers, community health workers, and community engagement specialists, involves the review of charts of hospitalized patients and/or patients at risk for exacerbation (eg, those with hospitalization or ED visits in specific seasons in years past). A structured review assesses both medical and social needs. Between January and September 2023, 80 patients were discussed, generally focusing on asthma history, comorbidities, adherence, triggers, and social determinants of health. More than 100 action items and responses across discussed patients included tailored medication management (eg, outreach to offer medication delivery), care coordination (eg, scheduling follow‐up visits, consulting subspecialists), and addressing health‐harming social needs. Relevant to such needs, we created and tested new referral mechanisms to connect children with asthma and substandard housing to resources available from partnered public health, legal aid, and social service agencies (eg, connections to sanitarians at local health and building departments or housing attorneys at our medical‐legal partnership).^36^, ^38^, ^39^ We track huddle action items identified and completed responses to gauge progress.

A final example innovation stems from an institutional priority to pursue social needs screening across our care settings. The Asthma Center (within the pulmonary division) recently rolled out assessments for social needs like housing exposures or instability, food insecurity, financial challenges, transportation insecurity, and caregiver mental illness. In the first 10 months of outpatient screening (November 2022–September 2023), 1180 Asthma Center patients have been screened; >50% have been positive for ≥1 need, with housing exposures/instability chief among them. Screens are paired with response algorithms. Example responses could include the housing connections described above or food from on‐site pantries in our primary care centers.^38^, ^39^ Social workers, care managers, care coordinators, and community health workers are also connected to patients as needed and desired.

## 4 DISCUSSION

Asthma is a complex disease with a complex history. Child asthma morbidity remains stubbornly prevalent. Moreover, unconscionable inequities characterize who experiences the worst that asthma offers. This is despite mounds of evidence, clear, though evolving, clinical guidelines, excellent clinicians, dedicated community‐based organizations, and caring families. Despite best efforts, care remains fragmented, without adequate visibility across care settings, and with limited accountability for optimal outcomes. This misalignment, and lack of meaningful organizational coordination, perpetuates suboptimal population‐level outcomes. We implemented ALHS in Cincinnati to overcome these challenges, equitably improve outcomes and reducing costs, and serve as a model for how we approach clinical care, community partnership, and research for other chronic conditions. Although it is too soon to gauge the impact on outcomes, early evidence of coordination and action is promising and motivating.

We built ALHS as a specific type of system‐level intervention, a collaborative learning health system. Pioneered at Cincinnati Children's, learning health systems have demonstrated measurable improvements across pediatric health conditions (eg, hypoplastic left heart syndrome,^21^ preterm birth,^40^ inflammatory bowel disease^17^) and community health outcomes (eg, inpatient bed days among children in at‐risk neighborhoods^7^). Other key organizational components, including interventions to improve situational awareness and decision‐making, have been effective in work relevant to inpatient safety^41^, ^42^ and the COVID‐19 pandemic.^23^, ^43^ A key, common mechanism of action relies on a networked organization, in which patients and families, clinicians, community partners, and more self‐organize and use shared resources to achieve shared aims.^16^, ^44^ As such, we expected that such methods, well suited to complex problems, could be effective when applied to the complexity of asthma.

Through ALHS, we aim to identify and scale healthcare‐based innovations that are evidence‐based and feasible. Opportunities abound for improvements across phases of care, from primary care to intensive care, especially in light of new asthma guidelines and persistent variation in practice.^45^ Several healthcare‐centric interventions have been studied and identified as effective in improving asthma outcomes. Example interventions prioritized in the ALHS design process include the following: (1) medications‐in‐hand at the time of discharge from the hospital and/or ED^4^, ^46^; (2) leveraged technology (eg, virtual visits, electronic inhaler monitoring) to promote self‐management and optimize medication adherence^47^; and (3) updating evidence‐based care pathways to align with new guidelines and decrease practice variation.^4^, ^48^, ^49^

Given the degree to which asthma morbidity is influenced by environmental factors, so too has ALHS sought to catalyze community‐based connections and innovations. We built ALHS, in part, to enable implementation of community‐based interventions that could improve outcomes at scale—and to do so in a coordinated fashion that that promotes learning across sectors. Priority areas, emergent from our design process, and from community‐based efforts proven effective both in Cincinnati and cities like ours, include the following: (1) asthma education and promotion of self‐management skills^50^; (2) care coordination across the system of care (eg, co‐management with school nurses and school‐based health centers^51^, ^52^); and (3) environmental trigger assessment and remediation.^36^, ^38^, ^39^, ^51^, ^53^, ^54^ We deemed approaches bridging clinical and community partners to address health‐harming challenges, like adverse housing conditions, as central, given mounds of evidence linking asthma severity to such unmet needs.^6^, ^55^, ^56^, ^57^, ^58^, ^59^

This experience report should be considered in the context of certain limitations. First, this paper describes our start and certainly not our end. In this report, we sought to depict the foundational work necessary for ALHS to flourish, and to achieve impact. We sincerely hope that a future publication will detail the degree to which ALHS achieves its bold, aspirational objectives, the newspaper headline we collectively envisioned. Second, our environmental scan unearthed multiple ways in which asthma is measured regionally and nationally. Direct comparison of regional measures to CDC benchmarks may, therefore, be inappropriate. Third, ALHS was created with and for youth with asthma living in our region. We acknowledge our approach may not generalize to other regions characterized by different risks and assets. That said, our learnings and approach are likely to be generalizable. Fourth, ALHS members agreed upon a primary measure related to hospitalizations. We did so because it was deemed valuable and measurable. There are certainly other measures of value not currently measurable at scale (eg, missed school, quality‐of‐life).

Despite these limitations, our early progress has positioned us for impact. To drive us forward, we continuously identify priority questions we must, collectively, answer. First, we see an urgent need for research into medical‐social care integration. How might we best respond to social needs identified in healthcare settings? What is the right “dose” of social need responses? What data are needed across sectors to drive collective action and accountability? Second, asthma is not a one‐size‐fits‐all disease. How might “precision medicine” be paired with “precision population health” approaches to right‐size care?^60^, ^61^ Indeed, certain patients may be more likely to benefit from certain medications (eg, inhaled corticosteroids, biologics). Certain populations (or neighborhoods) may be more likely to benefit from certain community‐based interventions (eg, housing code enforcement). Finally, we are only beginning to understand the power of learning health systems. Their application to complex, community‐based challenges and inequities requires further insight and investigation.

## 5 CONCLUSION

We need a different approach to asthma, one that enables the right care to be delivered at the right time, the right place, and in the right way to the right patients and populations. This approach to care should be complemented by investigators with the right expertise asking the right questions— curiosity driving the field forward. Learning health systems are uniquely suited to address asthma and its associated complexity, to enhance alignment, coordination, collaboration, and shared accountability.

## CONFLICT OF INTEREST STATEMENT

The authors state that they have no conflicts of interest to declare.

## Supporting information


**Table S1.** Prompts and questions used during qualitative data collection.
