# Supplementary material for: Building a regional pediatric asthma learning health system in support of optimal, equitable outcomes
Source: Learn Health Syst. 2023 Dec 11;8(2):e10403. doi: 10.1002/lrh2.10403 (PMC11019385; doi:10.1002/lrh2.10403)
Supplement: Supplementary file 1 — Table S1. Prompts and questions used during qualitative data collection. [file LRH2-8-e10403-s001.docx]

| **Prompts used during Group Level Assessments** |
| --- |
| **Community Assets**   - The most helpful community resources for kids with asthma and their families are… - If I could scale or grow one existing resource to help children with asthma, it would be... - Asthma treatment for children would be more accessible if… - The resources I wish were available for children with asthma are… - A service or support currently unavailable for children with asthma and their families is… - The best way for schools to support children with asthma is… - The best way for communities to support children with asthma is… - The best way for parents to support children with asthma is… - I’ve found ____ in the community most helpful when it comes to asthma.   **Family Experience with Care**   - In an ideal world, supporting children with asthma looks like… - The best ways for children with asthma to be supported in the clinic or hospital are… - Asthma gets in the way of daily life for families by… - Good asthma care is a struggle when… - The things that make asthma care for my child/children difficult are… - The biggest roadblock to improving asthma outcomes for children in Cincinnati is… - It is easiest for children with asthma to take their medication when… - In my opinion, the hardest part about caring for a child with asthma is… - _____ is working well when it comes to children’s asthma care.   **Situational Awareness**   - To manage asthma successfully, I need to know … - If there was an 'app’ to help me/families manage asthma, it would... - Care managers can be most effective with a child with asthma when… - I wish it were easier to find this information about asthma… - It would be easier to deal with asthma if I knew in advance that... - We will know that we are making improvements in asthma care here in Cincinnati when… - I would be better at predicting and avoiding bad asthma symptoms if I knew… |
| **Open-ended questions used for the focus group** |
| **Community Assets** – *identify assets and gaps, desired supports that do not currently exist, how communication flows (or does not) about family needs across resources.*   1. What do you think are the most helpful asthma resources for families? (inside and outside Cincinnati Children’s) 2. What resources do you wish were available to care for children with asthma in your community? What makes these hard to use currently? What would make these easier to use/access? 3. What services or supports related to your child’s asthma care do you most typically use in a month (…or what services do you refer families to most often)? What would make this/these service(s) easier to use?   **Family Experience with Care** – *identify how families experience the current care system, and outline what the idealized system would be (What would it look like if things were going well?).*   1. What does it look like for families on a good day of managing a child’s asthma? How about a bad day? 2. What makes it hard to get good asthma care? What makes it easy? 3. What is the trickiest/most difficult part of caring for a child’s asthma?   **Situational Awareness** – *identify ways that stakeholders could use up-to-date local data to make decisions and respond to needs.*   1. What do you consider the biggest trigger for asthma attacks in our community? 2. What is one thing that would make it easier to manage childhood asthma in Cincinnati? 3. What indicator would show you/how would you know that we are making improvements to asthma care here in Cincinnati? 4. How would you describe a child with asthma who is thriving? |
